# Hyaluronic-Acid-Based Organic-Inorganic Composites for Biomedical Applications

**DOI:** 10.3390/ma14174982

**Published:** 2021-08-31

**Authors:** Rebecca Sikkema, Blanca Keohan, Igor Zhitomirsky

**Affiliations:** Department of Materials Science and Engineering, McMaster University, Hamilton, ON L8S4L7, Canada; sikkemar@mcmaster.ca (R.S.); keohanb@mcmaster.ca (B.K.)

**Keywords:** hyaluronic acid, hydroxyapatite, gel, drug, biocement, bioglass, composite

## Abstract

Applications of natural hyaluronic acid (HYH) for the fabrication of organic-inorganic composites for biomedical applications are described. Such composites combine unique functional properties of HYH with functional properties of hydroxyapatite, various bioceramics, bioglass, biocements, metal nanoparticles, and quantum dots. Functional properties of advanced composite gels, scaffold materials, cements, particles, films, and coatings are described. Benefiting from the synergy of properties of HYH and inorganic components, advanced composites provide a platform for the development of new drug delivery materials. Many advanced properties of composites are attributed to the ability of HYH to promote biomineralization. Properties of HYH are a key factor for the development of colloidal and electrochemical methods for the fabrication of films and protective coatings for surface modification of biomedical implants and the development of advanced biosensors. Overcoming limitations of traditional materials, HYH is used as a biocompatible capping, dispersing, and structure-directing agent for the synthesis of functional inorganic materials and composites. Gel-forming properties of HYH enable a facile and straightforward approach to the fabrication of antimicrobial materials in different forms. Of particular interest are applications of HYH for the fabrication of biosensors. This review summarizes manufacturing strategies and mechanisms and outlines future trends in the development of functional biocomposites.

## 1. Introduction

Hyaluronic acid (HYH) is a natural polymer that plays a vital role in many physiological processes in humans and other organisms [1,2]. HYH is a major constituting part of the extracellular matrix of joints, eyes, and skin [2,3,4] as well as other parts of the human body [5]. HYH is a polysaccharide polymer that consists of repeating units of N-acetylglucosamine and glucuronic acid. As an existing molecule in the body, it is known to be biocompatible, non-toxic, non-immunogenic, non-inflammatory, and biodegradable [6]. Although high-molecular-mass HYH was first isolated in 1934 by K. Meyer and J. W. Palmer [7], a combination of HYH and sulfuric acid was first documented in 1918 by P. A. Levene and J. López-Suárez [8]. The discovery of HYH and methods for HYH synthesis have generated significant interest in biomedical applications of this biopolymer. These applications can typically be placed into the major categories of drug delivery, tissue engineering, and surface modifications. There are excellent reviews describing HYH applications for controlled drug delivery [9], treatment of inflammatory skin and joint diseases [10], wound dressing [11], fabrication of hydrogels for tissue engineering [12,13], and other applications in orthopedics, oncology, ophthalmology, and dermatology [14]. New manufacturing techniques, such as electrodeposition [15] and 3-D printing [16], have received particular attention in the literature for the fabrication of HYH films and scaffolds.

HYH is of significant interest for the fabrication of organic-inorganic composites for biomedical applications. Many natural materials, such as bone, are organic-inorganic nanocomposites. Natural composites combine the unique biomedical properties of biopolymers with properties of inorganic materials. Advanced functional properties of such composites also result from design features, such as multilayer and hierarchical structural organization, containing various building blocks at different dimensional scales [17]. The most prominent properties of natural organic-inorganic composites also result from chemical interactions of the organic and inorganic components and biomineralization phenomena. Therefore, the chemical properties of HYH are important for the design of organic-inorganic nanocomposites for biomedical applications.

Innovative studies during recent years have led to various applications of HYH for the fabrication of composite materials. The chemical structure of HYH facilitates the adsorption of this polymer on different inorganic surfaces. HYH has attracted attention for surface modification of inorganic particles and is used as a capping or structure-directing agent for the synthesis of inorganic nanoparticles and their dispersion [18,19,20]. Many new routes for the fabrication of films and scaffolds are based on the use of HYH-modified nanoparticles of inorganic materials [21]. Especially important are the colloidal properties and pH-dependent charge of HYH, which provide a basis for various colloidal and electrochemical manufacturing technologies [15]. Significant progress has been made in the application of HYH for the fabrication of advanced biocements [21]. Due to its unique gel-forming properties, HYH has generated significant interest for the fabrication of composite gels. HYH hydrogels can function as a matrix to incorporate both organic and inorganic substances to enhance tissue growth [22]. HYH and composites showed interesting biomineralization properties, which are currently under intensive investigation for various applications [23]. Previous reviews were mainly focused on pure HYH materials and HYH composites with different polymers, drugs, and other functional organic materials. This review addresses the need in the analysis of various composites containing different functional inorganic biomaterials, fabrication methods of organic-inorganic composites in different forms, applications of unique properties of HYH for the processing of inorganic biomaterials, microstructure development, and properties of organic-inorganic composites.

**Figure 1:**
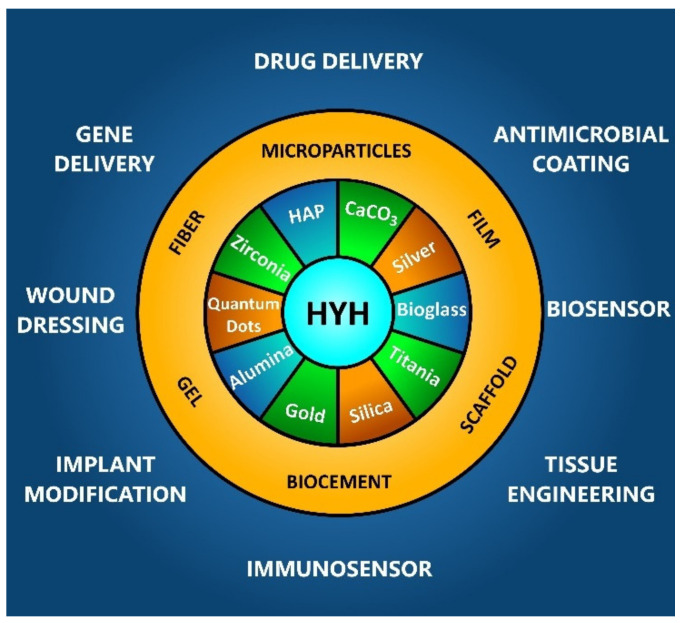
HYH-based organic-inorganic composites and their biomedical applications.

This review is focused on the functional properties of organic-inorganic composites containing HYH as an organic phase and their applications (Figure 1).

Our goal is to emphasize innovative ideas in biocomposite technology that are based on the unique properties of HYH. We describe recent advances in surface modification methods, which have enriched the science and technology of biomaterials. This review covers advanced functional materials for biomedical applications, such as scaffolds, gels, biocements, and materials for wound dressing. Another important area covered by this review is the application of HYH for the fabrication of composite films by electrochemical and colloidal methods. Special attention is paid to the synthesis and colloidal processing of functional inorganic materials using HYH as a capping and dispersing agent and their incorporation in the HYH matrix. Conceptually, new methods are described for the fabrication of composite materials for drug delivery and materials with antimicrobial properties. The unique microstructure and functional properties of HYH-based composites are key factors for new emerging developments in various fields of advanced biomaterials.

## 2. Structure and Functional Properties of HYH

**Figure 2:**
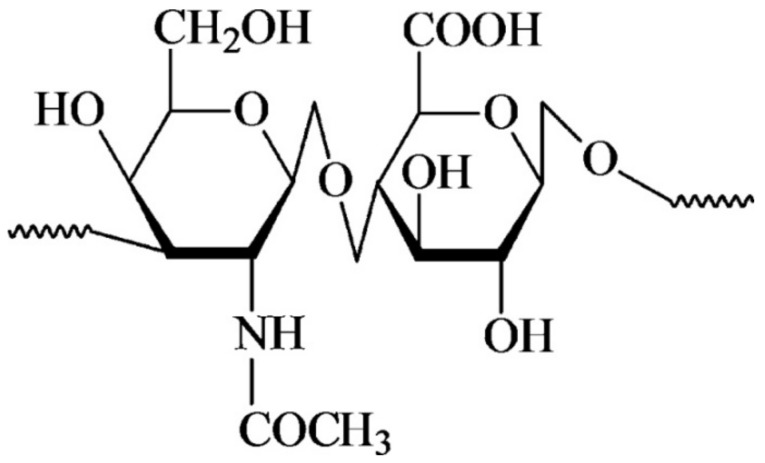
Chemical structure of HYH. Reproduced with permission [24]. Copyright Taylor & Francis 2009.

HYH is a natural biopolymer distributed widely throughout the human body. The HYH content in the human body was estimated as ~15 g for a body mass of 70 kg [14]. It is a biocompatible material well-suited for various biomedical applications. Figure 2 shows the chemical structure of HYH, consisting of alternating units of glucuronic acid and N-acetylglucosamine. In physiological conditions, HYH takes the form of a highly soluble sodium salt (HYNa).

HYH exhibits a pH-dependent charge and solubility in aqueous solutions. The anionic properties of HYH in solutions at pH > 3 are attributed to COO^-^ groups [25]. HYH exhibits a charge reversal at a lower pH at an isoelectric point of pH = 2.5. The positive charge of HYH below the isoelectric point results from the protonation of the –NH groups. HYH shows a gel-like behavior at pH = 2.5, and a reduction in the pH results in the dissolution of the gel [25] at pH = 1.6. HYH can be crosslinked with Cu^2+^, Zn^2+^, Ca^2+^, Fe^2+^, Fe^3+^, and other ions to form gels [26]. Important functional properties of HYH are governed by the amphiphilic spatial architecture of this polymer, which forms a two-fold helix with a hydrophobic part containing CH groups and the hydrophilic part of COOH and OH groups [27]. Such a chemical structure governs HYH interactions with different materials and imparts important properties for the fabrication of thin films, hybrid materials, and composites and facilitates various HYH applications as dispersing and capping agents for colloidal processing of materials.

HYH has many important physiological functions in the human body, which provide a platform for various applications. HYH is a remarkable natural lubricant that can be injected into the knee for the treatment of patients with knee osteoarthritis [9]. This biopolymer is also widely used for the treatment of other types of joint diseases [10]. HYH injection allows for a significant reduction in joint pain, synovial inflammation, and articular swelling [10]. Significant advances have been made in the development of different HYH-based materials for the treatment of inflammatory skin diseases [10].

HYH is an important biocompatible, hydrophilic, and gel-forming material for the treatment of dry eye disease. Moreover, the gel-forming properties of HYH are important for wound dressing and drug delivery applications [9]. Biocompatibility, biodegradability, and hydrophilic properties of HYH were utilized for the development of wound dressings in different forms, such as sponges, films, hydrogels, and membranes [11]. HYH is an efficient material for wound repair applications because it can improve wound healing by specifically binding to extracellular matrix proteins and thus stabilizing the extracellular matrix, mediating cell adhesion, and promoting growth factor action.

HYH is used as a marker in the diagnosis of cancers and liver diseases [14]. HYH has a remarkable ability to bind a large number of water molecules. As a result, HYH improves tissue hydration and their resistance to mechanical damage [14]. The hydrophilic properties of HYH were utilized for the modification of a contact lens surface in order to achieve enhanced water retention [28,29].

The remarkable adsorption properties of HYH facilitated the development of films and coatings for the surface modification of materials [30]. The pH-dependent charge and solubility of HYH were key factors for the development of electrophoretic deposition of HYH for surface modification of biomedical implants [15,24]. The strong interactions of HYH with other functional biomolecules were utilized for the electrophoretic deposition of composite films containing proteins, enzymes, antimicrobial agents, drugs, and other functional biomaterials [15]. The anionic properties of HYH facilitated the development of films using self-assembly techniques. Moreover, the colloidal properties of HYH facilitated the development of bioink techniques [16] and composite particles for medical treatments [31].

HYH has generated significant interest for the development of organic-inorganic composites for biomedical applications. Such materials combine functional properties of the individual components and exhibit new and advanced properties due to their synergetic effects. The primary research drive in this area is the need for advanced functional materials for biomedical implants, advanced gels, scaffolds, and biocements, coatings, and thin films for surface modification of biomaterials and biosensors. HYH provides a versatile platform for incorporation and retaining different sensitive biological elements for the fabrication of biosensors. The increasing interest in applications of HYH for the development of bone substitute materials resulted from the investigation of the complex hierarchical structure of bone and its mechanical properties. It was found [32] that polysaccharides form interfaces between organic and inorganic components in bones and govern the crystallization of hydroxyapatite nanoparticles. This important discovery [32] has generated a new wave of interest in the fabrication of bone substitute materials containing natural polysaccharides, such as HYH. The use of HYH as an organic component of bone substitute materials is very promising. It is known that HYH is a bioactive material, which promotes biomineralization of hydroxyapatite (HAP) and other calcium phosphates (CaPs) [33]. HYH was used as a template for the biomineralization of HAP [33]. It was shown that anionic carboxylic groups of HYH with chelating properties play an important role in the nucleation of HAP particles. The size, morphology, and Ca/P ratio of HAP particles can be controlled using HYH [33]. The bioactive properties of HYH were used for the surface modification of metals with CaP in simulated physiological solutions [34].

Due to its unique chemical structure and properties, HYH has been utilized for the synthesis and surface modification of advanced nanomaterials. HYH is an important capping and templating agent for the synthesis of biomaterials. It is important to note that many capping, dispersing, and templating agents for the synthesis of inorganic materials cannot be used in biotechnology due to their toxicity. Therefore, the use of HYH for the fabrication of organic-inorganic materials resulted in significant advances in biomaterials, which are described in the next sections of this review.

## 3. HYH–HAP and HYH–CaP Composites

### 3.1. Composite Gels

Composite HYH–HAP and HYH–CaP gels have been developed (Supplementary Materials, Table S1) with a variety of properties for many different biomedical applications [35]. The addition of HAP to the HYH gels improved the mechanical properties of the composite gels and reduced the water uptake, leading to a lower swelling capacity compared with the pure HYH gels [36,37,38,39]. Such changes were influenced by the degree of HYH crosslinking [38].

Glycidyl-methacrylate-conjugated HYH hydrogels were prepared [38] using a photo-crosslinking method, which improved their mechanical and biological properties. CaP particles were synthesized by in situ precipitation in the hydrogel matrix. The carboxyl group of HYH facilitated the fabrication of small and uniform CaP nanoparticles. The precipitation mechanism involved interactions of positively charged Ca^2+^ ions with the anionic carboxyl group of HYH, which acted as a nucleation site for the particle synthesis in subsequent reactions. Obtained HYH–CaP gels showed enhanced biocompatibility and promoted improved cellular responses compared with pure HYH gels [38]. 

**Figure 3:**
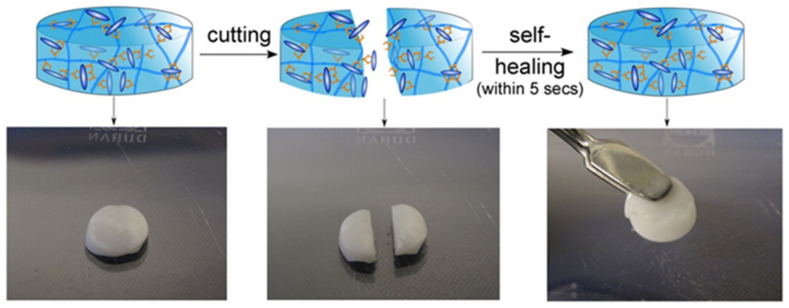
Self-healing behavior of non-covalently crosslinked HYH–CaP hybrid nanocomposites. Reproduced with permission [23]. Copyright Elsevier, 2014.

Bisphosphonate-functionalized HYH gel showed strong bonding to CaP and facilitated the fabrication of composite HYH–CaP gels, which exhibited self-healing properties (Figure 3) and good adhesion to enamel and hydroxyapatite [23].

Gels with a self-healing capacity present the unique possibility of injectability through a syringe. Extrusion through the syringe breaks the crosslinks, but the self-healing capacity reforms them after extrusion. The bisphosphonate groups grafted to HYH promoted biomineralization in vitro. The bone interactive properties of crosslinked gels were demonstrated in experiments on bone ingrowth [23]. These studies showed the benefits of strong non-covalent crosslinking, which involved interactions between CaP and the bisphosphonate groups bonded to the HYH.

**Figure 4:**
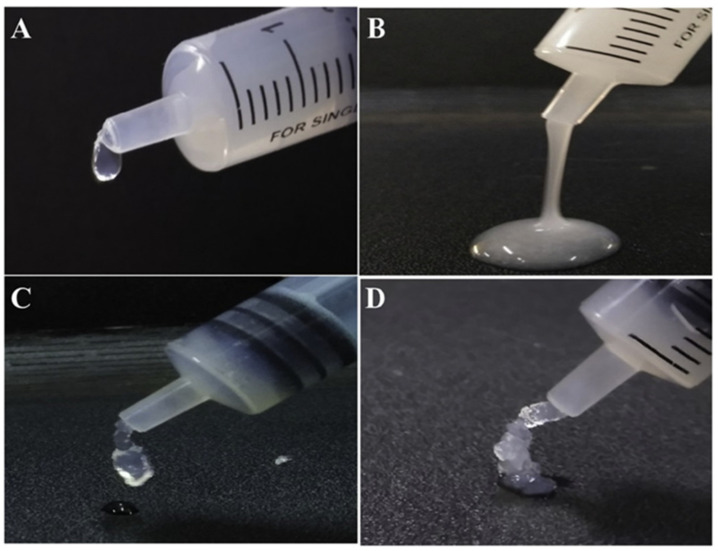
(**A**,**B**) The injectable HYH–HAP hydrogel and (**C**,**D**) the HYH–HAP–silk fibroin hydrogel form a three-dimensional gel scaffold. Reproduced with permission [39]. Copyright Elsevier, 2021.

The analysis of interactions between the carboxyl group on the sugar ring of HYH and the -OH group of HAP has driven the development of advanced gels (Figure 4), which exhibited improved mechanical strength and promoted bone tissue regeneration [39]. The hydrogel showed a porous structure and exhibited antimicrobial properties [39].

The investigation of the composite HYH–HAP gel’s microstructure highlighted the influence of HYH particle size and shape and particle size distribution on the gel properties [22,40]. Other investigations showed that gel pore size, porosity, and swelling decreased with increasing HYH concentration [41]. Many investigations were focused on the fabrication of injectable HYH–CaP and HYH–HAP hydrogels for the treatment of bone defects [41,42,43] and dermal filler applications [40,44]. It was found that HAP nanoparticles incorporated into HYH–HAP composites provided a physical barrier to enzymatic breakdown, which allowed for high resistance of the composite gel [44]. HAP nanoparticles were modified with zoledronate for the enhancement of the biomineralization properties of the HYH–HAP gels [45]. Surface functionalization of HAP nanoparticles with linear or branched polyethylenimine and arginine facilitated HAP dispersion and allowed for improved distribution of HAP in the HYH gel matrix [46], which was a prerequisite for a successful application in drug/gene delivery.

There is tremendous interest in the development of organic and inorganic crosslinking agents for HYH gels and analysis of their influence on the gel’s microstructure and properties [41,42,47,48]. Enhanced mechanical properties were achieved using a dual crosslinking technique, which combined photo-crosslinking and ionic crosslinking methods [47]. Ca^2+^, Ba^2+^, and Sr^2+^ ions exhibited the highest efficiencies in reinforcing the mechanical properties of HYH–CaP hydrogels [47]. HYH was combined with various biopolymers [35,48,49], such as cationic chitosan, anionic alginate, and other polymers, for the fabrication of CaP-containing hydrogels for drug delivery and bone regeneration applications. Co-polymers of HYH were developed for the fabrication of composite gels containing HAP for enhanced bone regeneration [50]. An important strategy was based on the synthesis of pyrogallol-conjugated HYH [51]. The chelating properties of the pyrogallol ligand facilitated the fabrication of adhesive hydrogels containing HAP and other inorganic minerals for orthopedic applications [51]. The chelating galloyl ligands promoted intermolecular interactions of the HYH molecules.

**Figure 5:**
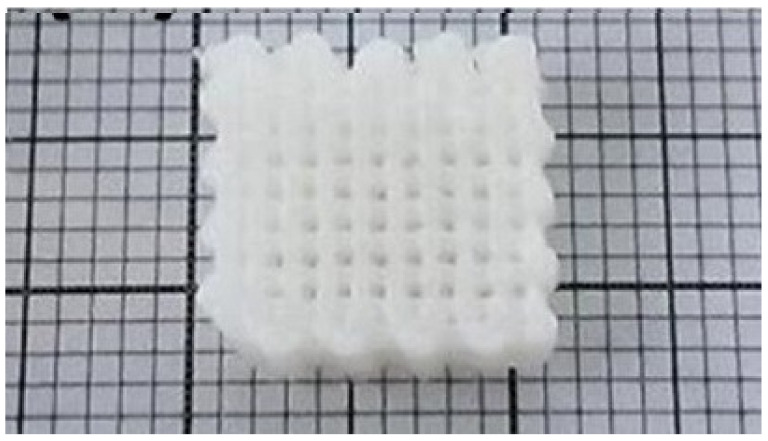
Printed grid structure based on HYH–HAP bioink. Reproduced with permission [36]. Copyright IOP Publishing, 2017.

HYH–HAP hydrogels also give rise to applications as inks for bioprinting. Wenz et al. [36] developed HYH–HAP gels using the photoinitiator lithium phenyl-2,4,6-trimethylbenzoylphosphinate. The HAP nanoparticles found in the gel primarily exhibited a spherical morphology, although some demonstrated a plate or rod morphology. These nanoparticles were non-covalently incorporated into the gel and formed a viscous phase. It was found that a gelling point of 21.5 ± 0.31 °C is ideal for the smooth printing (Figure 5) of this bioink [36].

### 3.2. Composite Films and Coatings

#### 3.2.1. Electrophoretic Deposition

Electrophoretic deposition (EPD) involves electrophoresis of charged macromolecules or particles and film formation at the electrode surface [52,53]. Composite films can be formed by co-EPD of polymers and inorganic materials [54]. It was found [24] that pure HYH films can be deposited by EPD. Moreover, composite HYH–HAP and HYH–CaP films can be formed (Supplementary Materials, Table S1) by the co-EPD method [24,55]. The EPD mechanism [24,56] of HYH films is based on the dissociation of sodium hyaluronate (HYNa) into HY^-^ and Na^+^:

HYNa → HY^−^ + Na^+^ (1)

The electric field provides the electrophoretic force moving HY^−^ towards the anode, where the pH is low due to water decomposition, resulting in free H^+^ ions:

2H_2_O → O_2_ + 4H^+^ + 4e^−^ (2)

The negatively charged HY^-^ is neutralized at the electrode by the free positively charged H^+^, resulting in the HYH film’s formation [24]:

HY^−^ + H^+^ → HYH (3)

HY^-^ can be adsorbed onto the surface of HAP, thereby providing an electrical charge for the HAP particles, allowing them to move in the electric field [24]. Moreover, the adsorbed HY^-^ provided electrosteric stabilization of HAP nanoparticles in the suspensions. HAP was then trapped in the HYH matrix, forming a composite HYH–HAP film [24]. The major benefit of this technique is that it offers room temperature processing, and the developed films are relatively uniform in structure [55,57,58]. The method enabled the formation of composite films of different thicknesses in the range of 0.1–100 μm [24]. The deposit composition can be varied by the variation of the HAP concentration in the HYNa solutions [55]. Composite films showed corrosion protection of stainless steel substrates in Ringer’s physiological solutions [24].

**Figure 6:**
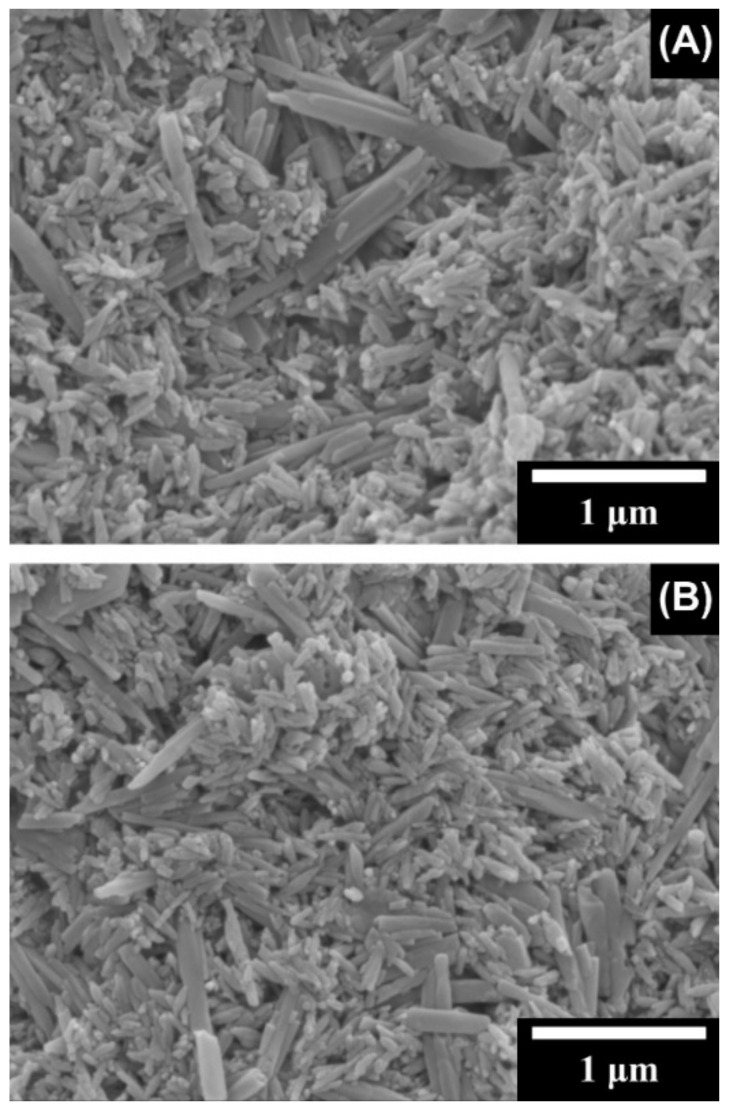
SEM images of composite films prepared by EPD from 1 g L^−1^ HYNa solutions, containing 1 g L^−1^ HAP and (**A**) 0.3 and (**B**) 0.6 g L^−1^ halloysite nanotubes. Reproduced with permission [57]. Copyright Elsevier, 2014.

The EPD technique can be utilized to co-deposit HYH and HAP with other functional materials, such as halloysite nanotubes for drug delivery applications or bioglass for the development of implants with enhanced bioactive properties. Following this mechanism, HYH–HAP films containing halloysite nanotubes (Figure 6) [57], carbon nanotubes [58], bioglass [55,56], and graphene oxide [59] have been developed. Each of these films was continuous and crack-free. Additionally, the film had little agglomeration of the inorganic particles [57,58]. The films were deposited as monolayers or multilayers, containing different individual layers [58].

#### 3.2.2. Other Deposition Techniques

Huang et al. [60] developed (Supplementary Materials, Table S1) a new mechanism of HYH and chitosan (Chit) deposition by a layer-by-layer (LBL) technique onto nanocellulose–HAP layers, which were formed by the precipitation of HAP on nanocellulose from solutions containing Ca^2+^ and PO_4_^3−^ ions. The anionic HYH and cationic Chit were added by alternately dipping the matrix into solutions of these polymers, starting with Chit. The developed films were highly hydrophilic and became more hydrophilic with an increasing number of individual layers. The addition of HYH increased the mechanical strength. Greater numbers of film layers decreased the hardness of the film. The cell viability increased with the LBL deposition on the surface, as there is an increased surface area and surface affinity following the LBL assembly [60].

HYH can be adsorbed onto HAP or CaP surfaces to create a polyelectrolyte-based composite coating [61,62,63]. This adsorption occurs due to the partial dissolution of either the HAP or CaP surface layers [64]. The released ions complex with HYH and reprecipitate at the sample surface, forming a strongly attached HYH layer to the surface. The composite films showed antibacterial properties for Gram-positive and Gram-negative bacteria [62,65].

### 3.3. Scaffolds

Scaffolds have been found (Supplementary Materials, Table S1) to be extremely beneficial in tissue engineering to create an environment for cells to proliferate and differentiate [66]. HYH–CaP scaffolds have been shown to create this environment, particularly for bone tissue [66,67]. The formation of hybrid scaffolds allowed for reduced swelling and degradation rates [66]. An HYH–gelatin hydrogel system was combined with CaP granules and crosslinked. The porosity of the gel increased the surface area compared with the pure CaP granules, giving a final porosity of 56%. The increased surface area encouraged cell and protein attachment, which is highly beneficial for tissue engineering. Due to the network structure of the gel, the addition of HYH and gelatin to the system increased the scaffold’s mechanical strength. Additionally, the gel in the scaffold degraded at a much slower rate than the pure HYH–gelatin gel. There was increased cell growth and viability on the composite scaffold compared with cell growth on CaP granules. In the HYH–gelatin–CaP scaffold, the cells were distributed throughout the scaffold structure and greatly increased bone growth [66]. It was found that the uniform distribution of CaP throughout the HYH-based scaffold improved the scaffold’s stiffness and mechanical properties [68]. Polymer crosslinking improved the mechanical and physiological stability to the scaffold [68]. Kaczmarek et al. [69] developed an in situ CaP precipitation technique and showed that CaP particles adhered to the scaffold’s surface. The scaffold’s density and porosity increased with increasing HYH content [69].

HYH was also combined with other biopolymers in order to improve porosity and biocompatibility [67,70,71,72,73]. HYH–Chitosan–Collagen–HAP scaffolds have been developed with a porosity of over 90%. In this approach, HAP was precipitated onto the previously crosslinked scaffolds and was homogeneously distributed throughout the scaffold. The amount of crosslinking of HYH affected the stiffness of the scaffold. The use of HYH in the scaffold improved the thermal stability of the scaffold. Cells were viable on the scaffolds and showed improved viability when HYH was in the scaffold compared with pure polymeric scaffolds [67]. Nano-HAP was introduced into hydrogel HYH/poly(γ-glutamic acid) scaffolds due to its natural ability to promote bone regeneration [74]. Moreover, HAP was found to significantly increase the compressive strength of the porous scaffolds [72,75]. It has been demonstrated [76] that the addition of HAP to the polymer phase reduced the degradation rate of the polymer-based scaffold materials. Increasing interest has been generated in the application of biphasic calcium phosphate (BCaP), which consists of HAP and tricalcium phosphate phases [73]. BCaP is considered to be an ideal bone substitute material due to its controllable degradability and high rate of bioresorption [73]. The anionic HYH was combined with cationic chitosan to form a complex, which was crosslinked and used for the fabrication of a composite containing HAP particles [77]. The obtained scaffold material showed excellent properties for cell penetration, growth, and proliferation, which are promising for bone repair application [77]. A biphasic osteochondral scaffold material has been developed [78], which was composed of a HYH/atelocollagen chondral phase and a HAP/CaP osseous phase for repairing osteochondral defects. Many investigations focused on the development of advanced crosslinking agents, which represent an important factor controlling the mechanical properties of scaffolds and their biocompatibility [79,80]. Significant advances were achieved by the development of advanced fabrication techniques, which facilitated the control of porosity [81,82]. A unique porous microstructure and unique properties of scaffolds for dental applications were achieved using a freeze drying technique [81].

### 3.4. Biocements

Calcium phosphate cements (CPCs) containing HYH (CPC-HYH) have generated significant interest (Supplementary Materials, Table S1) for various biomedical applications [83,84,85,86]. CPC setting involves reactions of mixed CaP phases with a liquid. CPC-HYH have been fabricated by the incorporation of HYH microparticles into CaP powder to form a paste that can be hardened into a cement [87]. The greater the amount of incorporated HYH microparticles, the more brittle the cement became and was characterized by a powdery surface [87].

The HYH microparticles were incorporated into the cement to make the cement more porous and, therefore, make it more degradable, as the highly interconnected structure facilitates its degradation. The addition of platelet lysate proteins was used to further increase the macroporosity. The HYH microparticles found in this cement have a very low degradability due to their crosslinking and encasement in the cement surface [87]. In another investigation, a cement paste was used that contained CaP crystals surrounded by a liquid phase [88]. HYH facilitated water retention, thus increasing the liquid phase’s viscosity and increasing the resistance to cement disintegration [88]. The use of medium- and high-molecular-weight HYH additives enhanced the cement cohesion [88]. The properties of CPC-HYH were influenced by chemical interactions of the carboxylic groups of HYH and Ca ions on the surface of the CPC phase [88]. Investigations revealed the influence of the HYH phase on the CPC setting time and injection behavior [88,89].

The addition of HYH to CPC to form a paste greatly increased the injectability of the paste [90]; however, there is very little dependence of this increase on the concentration of HYH incorporated. HYH promoted the formation of HAP from the CaP, as it accelerated the dissolution of CaP and promoted ion exchange to accelerate the HAP crystal growth from the greater supersaturation degree of both the Ca^2+^ and PO_4_^3−^ ions [90]. The addition of HYH increased the compressive strength of the cement due to the great anchoring strength of HYH in the cement matrix and the improved cohesiveness resulting from the HYH addition. This increase in strength is also due, in part, to the formation of HAP in the cement. When implanted in vivo, the addition of HYH allowed for the formation of much more new bone with highly dense mineralization compared with HAP cements [90].

Another interesting strategy was based on the use of composite HYH–alginate microbeads, which were incorporated into the CPC matrix and facilitated the cement’s injectability [91]. The addition of microbeads in CPC did not vary the setting time and compressive strength significantly. Another strategy has been developed for the fabrication of injectable CPC-HYH for bone repair or reconstruction applications [92]. In this case, an HYH network spreading all over the paste was formed, which resulted in good maintenance of the cement’s shape [92]. The chelating reactions of hyaluronate and Ca^2+^ ions on the HAP surface promoted HAP crystal interlocking. The injectability, setting time, and mechanical strength were varied by changing the HYH phase content in the composite cement. HYH was functionalized with bisphosphonate [93] to enhance polymer bonding to the inorganic phase, which facilitated the development of a cement with improved cohesion and mechanical properties.

Investigations in this area also reported the application of HYH–tyramine (Tyr) conjugates for the fabrication of a HAP–HYH–Tyr cement, which was hardened by enzyme-mediated coupling of Tyr moieties [94]. This cement offered the benefit of reduced heat release during the setting process compared with other cements, such as polymethylmethacrylate bone cements. Other advantages were attributed to tunable mechanical properties, a relatively fast setting time, and reduced swelling.

### 3.5. Composite Particles

Composite HYH–CaP and HYH–HAP particles (Supplementary Materials, Table S1) are under development for drug delivery applications [95]. HYH is an important material for targeted drug delivery systems in chemotherapy [96]. The literature has shown that HYH nanoparticles, mineralized by CaP, can be used as a robust carrier of the anticancer drugs [97,98]. Such particles were designed for controlled drug release and allowed for an enhanced anticancer effect [96]. HYH was modified with dopamine to enhance the adsorption of the modified polymer on CaP particles [99]. In this approach, the hydrophilic HYH backbone acted as a protective shell that prevented excessive growth of CaP nanoparticles and served as a targeting moiety for CD44 cancer cells [99]. In another investigation [100] of HYH–HAP composite nanoparticles, it was demonstrated that HYH acted as a tumor-targeting active ligand to bind the CD44 receptors on the tumor cell surface, and HAP allowed for drug loading and delivery of doxorubicin to the tumor cells. The specific binding between hyaluronan and CD44 protein was used for the application of composite particles containing HYH and doped HAP for tumor-specific bioimaging [101].

HYH-functionalized pH-responsive CaP nanoparticles showed a promising performance for application in tumor targeted therapy [102]. Surface modification of HAP with HYH–chitosan multilayers facilitated the fabrication of hollow hybrid microparticles with controllable size, wall thickness, and drug delivery properties [103]. In this strategy, the drug release of the hollow hybrid microparticles was pH-dependent due to tunable electrostatic interactions and permeability of the HYH–chitosan polyelectrolyte multilayers at different pH values and the dissolution of the HAP hollow core under acidic conditions [103]. HYH–chitosan-modified HAP particles were also loaded with Au nanorods, which prevented drug leakage and imparted an excellent near-infrared (NIR)-triggered drug release property [104]. A high drug loading efficiency was reported for composite particles containing an HYH/polyethylene glycol polymer shell and a nano-HAP core [105]. The fabrication method developed in this investigation facilitated the synthesis of particles with enhanced chemical stability and biocompatibility [105].

## 4. HYH–Bioceramic and HYH–Bioglass Composites

### 4.1. Silica

**Figure 7:**
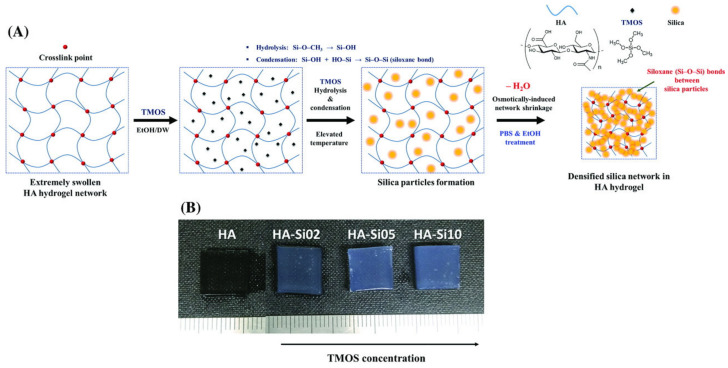
(**A**) Schematic illustration of the fabrication and (**B**) an optical image of HYH–silica nanohybrid hydrogels. Reproduced with permission [106]. Copyright Wiley, 2018.

Composites of HYH and silica (SiO_2_) have been developed (Supplementary Materials, Table S1) as hydrogels or films for many applications, such as drug delivery and tissue engineering. One promising manufacturing strategy [106] involved taking advantage of sol–gel synthesis and freeze-drying to produce the interconnected microporous structure of the HYH-SiO_2_ gels. Increasing the silica content in the gel formed more dense gels. The rigid silica network was found throughout the gel matrix and developed a nano-roughened surface on the gel (Figure 7). The developed gels were highly flexible and could be put under high levels of deformation. The networked silica structure in the gel transferred the load, which allowed the gel to resist the strain. Additionally, the silanol groups and HYH were able to form hydrogen bonds, thereby further enhancing the mechanical properties. Cells were highly viable on these gels [106].

New gelling and crosslinking strategies have emerged as convenient and rapid methods for incorporating advanced functionality into HYH–silica gels [107,108]. Of particular interest is a new approach [107], which was based on the crosslinking of the polysaccharide chains by a 3-D siloxane organic-inorganic matrix using a sol–gel method. Polycondensation and crosslinking reactions, as well as drying conditions, have been optimized for the development of advanced organic-inorganic composites [107]. Crosslinked HYH–silica nanohybrids with homogeneously distributed silica throughout the HYH matrix were obtained [109]. The crosslinking of the gel prevented the tight packing of HYH chains, so the gel had a larger volume compared with the non-crosslinked gels. The larger amounts of silica in the gel reduced swelling by decreasing the hydrophilicity of the gel due to the lack of silanol groups available to bind with water molecules. The higher degrees of crosslinking found in the gels with high amounts of silica also reduced swelling [109].

Among the interesting examples of HYH–silica gel applications is the development of biosensors [110]. HYH–polyethyleneimine gels were loaded with ruthenium (II) chloride hexahydrate-doped silica nanoparticles to measure the in vivo concentration of hyaluronidase (HYHase). HYHase decomposed the hydrogel, which allowed for the release of the Ru–silica nanoparticles, and their electrochemiluminescence signal was measured to find the HYHase concentration [110].

HYH–SiO_2_ hydrogels have been developed for drug delivery. SiO_2_ particles can be loaded with a variety of drugs, such as Rose Bengal (RB) [111] or doxorubicin [112]. The amount of drug released increased with the increased amount loaded into the gels. The normalized release amounts of the drugs were greater when they were directly loaded onto the silica particles rather than directly loaded into the gel. SiO_2_ microparticles have high loading amounts of RB, as the microparticles have a large surface area. During the drug release, RB first diffuses from the silica particles into the HYH matrix, then diffuses from the HYH matrix into the surrounding media. As the RB diffuses from the gel, the gel swells as water molecules can then be adsorbed by the gel [111]. To release doxorubicin from the hydrogel, HYHase breaks down the gel in vivo, which allows for the release of doxorubicin from the SiO_2_ nanoparticles. Near-infrared radiation can be used to further degrade the hydrogel, allowing doxorubicin to be released more easily [112]. Injectable hydrogels for surgery have also been reported through the use of HYH and silica [113].

**Figure 8:**
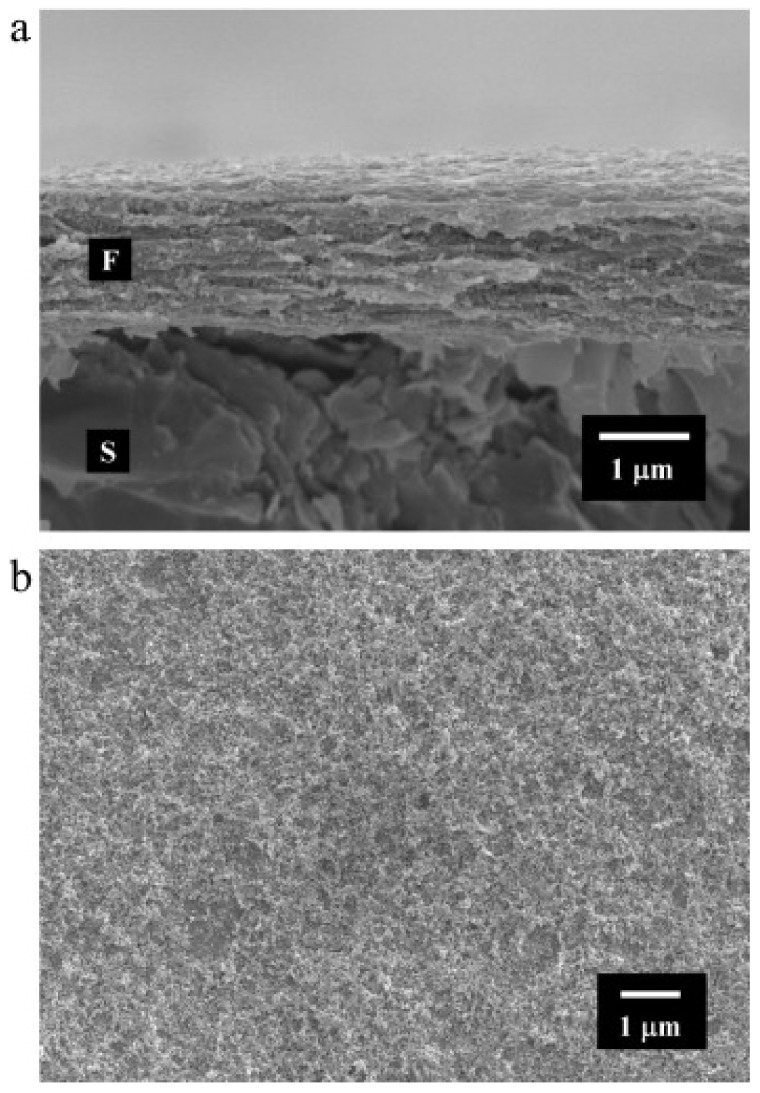
SEM images of (**a**) a cross section (fracture) and (**b**) the surface of a film prepared by EPD from a 0.65 g L^−1^ silica suspension containing 0.5 g L^−1^ HYNa on a graphite substrate (F—film, S—substrate) at a constant voltage of 20 V. Reproduced with permission [114]. Copyright Elsevier, 2011.

HYH–SiO_2_ films have been developed by anodic EPD [114]. The developed films were dense and crack-free, showing a uniform distribution of silica nanoparticles (Figure 8). The amount of material deposited increased with increasing time and voltage [114].

Increasing the amount of silica in the solution allows the silica nanoparticles to cover a greater surface area of the film, increasing the surface roughness. The silica nanoparticles have also been shown to increase the mechanical properties of the gel. The solution is stable in suspension due to hydrogen bonding between HYH and silica [115]. The silica particles become negatively charged due to the adsorption of HY^-^ onto the surface of the silica particles. This adsorption then stabilizes the silica suspensions [114]. The incorporation of silica into the HYH coatings resulted in enhanced mechanical stability and increased lubrication properties [115].

HYH–poly(6-lysine)–silica films have also been developed by polyelectrolyte multilayer formation followed by immersion of the film into a solution containing a silica precursor of sodium silicate [116]. The multilayers were formed by dip coating, and the layer thickness grew exponentially. The addition of the inorganic component dispersed homogeneously throughout the material increased the film stiffness. Additionally, it induced osmotic stress and swelling in the film [116]. Researchers have employed LBL self-assembly for the fabrication of mesoporous silica nanoparticles and HYH–cyclodextrin films, which were constructed to load the fluorescent dyes and peptides. The release rates of these molecules were varied by the variation in the number of layers in the multilayer films [117,118].

### 4.2. Titania

The material system of HYH–titania (Supplementary Materials, Table S1) offers many benefits for various biomedical applications due to the unique functional properties of the individual components. Titania nanotubes were used as drug carriers for the fabrication of HYH–titania implants for controlled drug release [119]. This material system facilitated uniform drug release and exhibited enhanced biocompatibility, promoted osteogenesis, and showed an anti-osteoclastogenesis ability [119]. In another investigation, titania nanofibers were combined with methacrylated HYH for the deposition of composite films on the polyetheretherketone surface for the fabrication of implants with enhanced biocompatibility and osteogenic activity [120]. Mu et al. loaded titania nanotubes with raloxifene and modified the nanotubes with HYH–alendronate for controlled raloxifene release and enhanced osseointegration [121]. Deferoxamine-loaded titania nanotubes were modified with HYH–gentamicin and chitosan layers for the fabrication of films with antibacterial and antifouling properties on Ti implants [122]. Enhanced adhesion and antimicrobial properties were obtained using catechol-modified HYH–chitosan multilayers containing titania nanotubes loaded with an antibacterial drug [123].

HYH–titania composites showed improved antifungal activity compared with the individual components [124]. It was shown that such composites are promising for applications in food, biomedical, environmental, and pharmaceutical industries [124]. Another important property of HYH–titania composites is their anticancer activity, which can be enhanced by the optimization of the composite’s design, microstructure, and composition [125]. The progress achieved in the surface modification of biomaterials with HYH–titania has generated interest in the development of film deposition techniques. The ability to form HYH–titania films by EPD opens up new opportunities for the surface modification of metallic implants [114]. Another strategy involved the formation of multilayers by self-assembly of anionic HYH and cationic biopolymers. Such multilayers can entrap Ti precursors and facilitate the formation of titania nanoparticles in the biopolymer matrix [116]. Carbonized HYH–titania composites were used for the development of advanced biosensors for visualization of the cell adhesion interaction with the surface and detection of cancer cells [126]. It was demonstrated that cell interaction with a carbon dot/Cu^2+^-modified HYH–titania composite irradiated under visible light resulted in changes in electrical resistance and fluorescence intensity, which could be recorded by an optical microscope and a wireless device.

### 4.3. Calcium Carbonate, Alumina, and Zirconia

**Figure 9:**
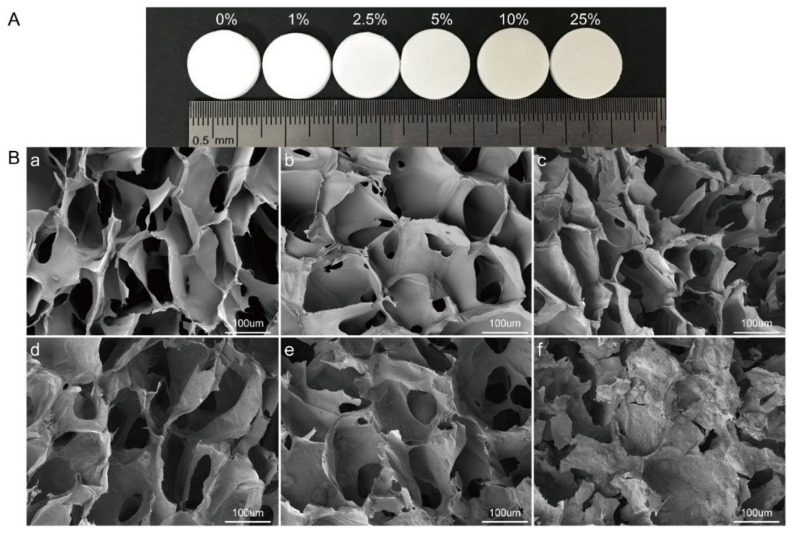
(**A**) Photograph of HYH–chitosan–CaCO_3_ scaffolds with different proportions of CaCO_3_. (**B**) Pore morphology of HYH–CaCO_3_ scaffolds imaged by SEM. (**a**–**f**) show the morphology of scaffolds with 0%, 1%, 2.5%, 5%, 10%, and 25% CaCO_3_, respectively. Reproduced with permission [129]. Copyright Elsevier, 2020.

HYH–CaCO_3_ composites (Supplementary Materials, Table S1) have generated interest for the fabrication of biomedical scaffolds for the treatment of both chondral and osteochondral defects in humans [127]. It was found that impregnation of CaCO_3_ with HYH enhances the scaffold’s regenerative potential [127]. The interest in the application of HYH–CaCO_3_ scaffolds has generated research efforts on the development of advanced manufacturing techniques [128]. It was found that HYH exerts an influence on the shape of CaCO_3_ crystals, which were formed in the presence of HYH. The HYH matrix acted as a template for the growth of the CaCO_3_ crystals. It entrapped the Ca^2+^ ions due to the complexation between HYH and Ca^2+^ ions and facilitated the crystal nucleation [128]. The addition of CaCO_3_ to HYH resulted in improved mechanical properties [129]. The porous scaffolds (Figure 9) with high CaCO_3_ content were also beneficial for cell proliferation and differentiation [129].

CaCO_3_ is a promising material for protein encapsulation and release. The high protein entrapment efficiency of CaCO_3_ particles was reported in [130]. The interactions of proteins with CaCO_3_ particles were investigated [131] and used for the development of HYH-based composites containing CaCO_3_ particles loaded with proteins for controlled protein release.

HYH-functionalized alumina hollow particles were developed for controlled drug delivery for liver cancer therapy [132]. In this approach, HYH allowed for controlled drug release [132]. A HYH–alumina membrane has been developed for applications in immunosensors for bacterial pathogens [133]. In this approach, HYH reduced the steric hindrance of immobilized antibodies, making them readily accessible to the target bacteria. HYH was used to maximize the ratio of the sensor signal to the background noise and improve bacterial accessibility to the antibodies. This allows the immunosensor to have a much higher capture efficiency and become much more sensitive [133].

Zirconia surfaces were coated with HYH hydrogels [134]. These hydrogels were loaded with growth factors or proteins for their release to aid in new bone formation. The amount of the factors or proteins loaded has a significant impact on their release rates. The addition of the HYH gel reduced this initial burst release and allowed for a much more sustained release of the factors and proteins. Moreover, the addition of HYH increased the hydrophilicity of the surface, leading to a lower contact angle. The ability of the gel to swell enabled the adsorption of increased amounts of proteins to the surface. Due to this increased protein adsorption, improved cell proliferation and differentiation on the surface were observed [134]. HYH was used to tune the functionality of doped zirconia for the repair of bone defects, and an enhanced bone regeneration rate was achieved.

### 4.4. Bioglass

HYH–bioglass composites (Supplementary Materials, Table S1) have generated interest for the fabrication of bioactive scaffold materials, pastes, and coatings [56,135,136,137]. HYH–dopamine conjugates showed enhanced adhesion on inorganic surfaces due to the chelating properties of the catechol group of dopamine [138]. The adhesive properties of such conjugates were combined with antimicrobial and bioactive properties of Ag-doped bioglass nanoparticles for the fabrication of multifunctional biocomposites, which were prepared by LBL assembly for application in orthopedic implants [138]. Catechol-modified HYH was also combined with catechol-modified chitosan for the fabrication of advanced multilayer bioactive films containing bioglass or Ag-doped bioglass [139,140,141]. Due to the use of catechol-modified polymers, the films showed enhanced adhesion to the substrates [139]. Additionally, Moreira et al. reported that incorporation of catechol groups into the films showed a significant increase in the films’ stiffness and lower swelling [140]. The use of Ag-doped bioglass facilitated the development of composites with antimicrobial properties [141]. Bisphosphonate-functionalized HYH has been developed and used to achieve enhanced interaction of the polymer with bioglass [142]. The obtained composite hydrogel showed advanced bioactivity and self-healing properties for bone-tissue engineering applications [142]. Composite HYH–bioglass films were prepared by EPD for surface modification of metallic implants [55,56]. The deposition method involved charging of bioglass by adsorbed HY^-^ species, electrophoresis of charged bioglass particles, protonation and discharge of HY^-^ in anodic reactions, and formation of composite HYH–bioglass films on the anodic substrates. Manferdini et al. [143] analyzed the bioactivity of HYH–bioglass scaffolds under different conditions and showed that fine pH control is an important factor for biomineralization. The composite scaffolds showed a promising performance for bone repair. Injectable biocomposites have been developed based on sol–gel-derived bioactive glass nanoparticles and HYH [144]. It has been demonstrated that obtained biocomposite pastes containing dispersed bioglass nanoparticles could be completely injected through a standard syringe using a low compressive load for the treatment of hard and soft tissues. The investigation of pastes in which the liquid phase was HYH and 10% sodium alginate solution revealed a better apatite formation ability and better washout resistance than that made of HYH alone [137].

## 5. HYH Composites Containing Silver, Gold Nanoparticles, and Quantum Dots

### 5.1. Silver

**Figure 10:**
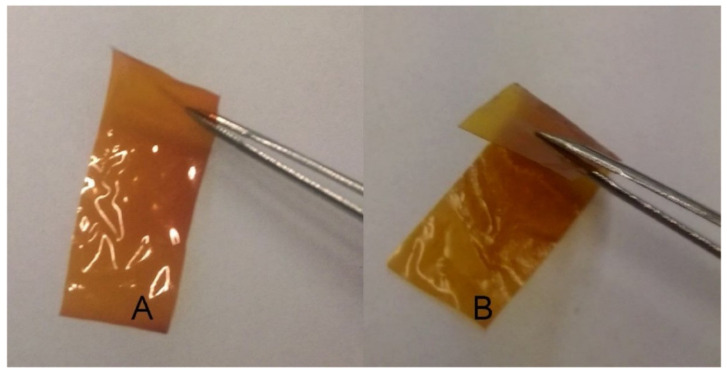
(**A**) HYH–Ag and (**B**) HYH–Ag–lecithin foils. Reproduced with permission [152]. Copyright Elsevier, 2016.

Significant interest has been generated in the fabrication of HYH–Ag-based composites (Supplementary Materials, Table S1) for biomedical applications [145,146,147]. The addition of Ag to HYH imparts antimicrobial properties to the composites [148]. An antimicrobial sponge composed of chitosan, HYH, and Ag nanoparticles (AgNPs) was prepared for wound dressing applications [149]. HYH–alginate hydrogels containing AgNPs exhibited valuable antimicrobial properties for treating infected wounds [150]. AgNPs were embedded in HYH/polycaprolactone nanofibrous membranes to prevent peritendinous adhesion and bacterial infection after tendon surgery [151]. Free-standing HYH–AgNP foils (Figure 10) for biomedical applications were prepared, and it was found that the properties of such films can be improved by adding lecithin as a natural surfactant [152,153]. In this approach, the use of crosslinking agents can be avoided, and the foils contained only natural components [152].

**Figure 11:**
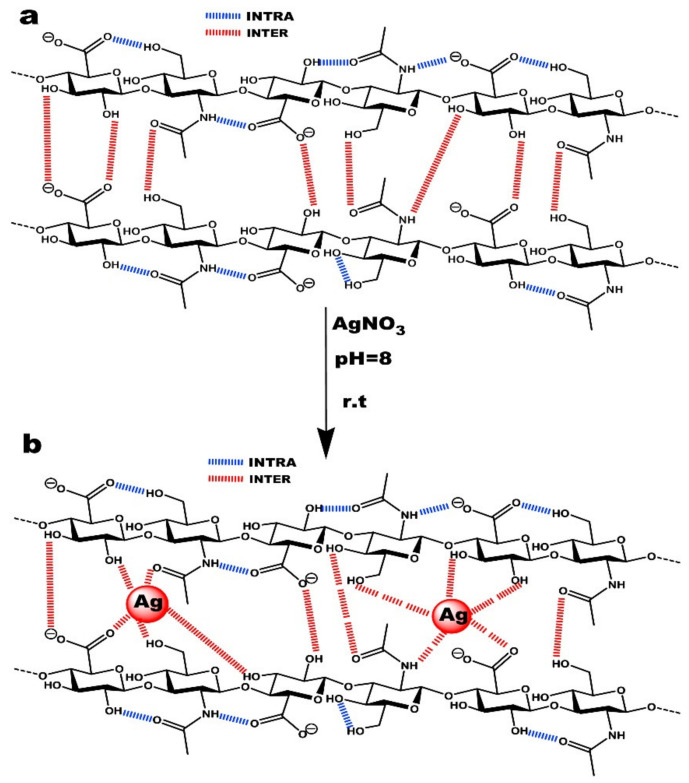
(**a**) Intra/intermolecular hydrogen bonds between hyaluronan chains and (**b**) the interaction mechanism between hyaluronan functional groups and silver nanoparticles. Reproduced with permission [155]. Copyright Elsevier, 2017.

HYH was used not only as a component of composite materials but also as a reducing agent for Ag^+^ ions, which facilitated the synthesis and dispersion of AgNPs [154]. It was suggested that hydroxyl, carboxyl, and acetamide groups of HYH (Figure 11) can be involved in the formation and stabilization of AgNPs [155].

**Figure 12:**
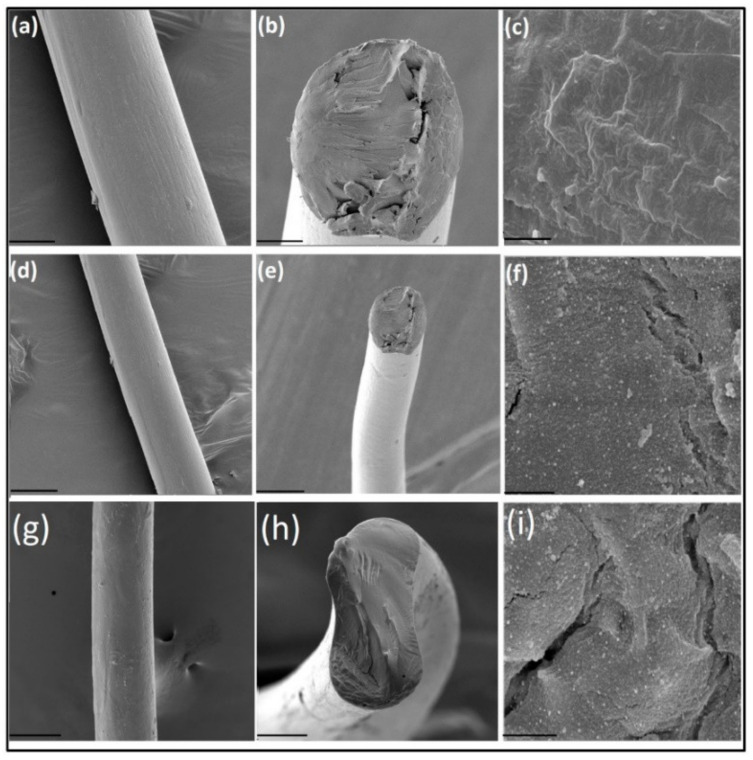
Scanning electron micrographs of plain HYH fibers (**a**); cross section of HYH fibers (**b**); HYH fibers at high magnification (**c**); a HYH/Ag-NPs-1 mg fiber (**d**); cross section of the HYH/Ag-NPs-1 mg fiber (**e**); the HYH/Ag-NPs-1 mg fiber at high magnification (**f**); a HYH/Ag-NPs-2 mg fiber (**g**); cross section of the HYH/Ag-NPs-2 mg fiber (**h**); the HYH/Ag-NPs-2 mg fiber at high magnification (**i**). Scale bars for (**a**,**d**,**g**), 100 μm; (**b**,**e**,**h**), 50 μm; and (**c**,**f**,**i**), 2 μm. Reproduced with permission [155]. Copyright Elsevier, 2017.

Composite HYH fibers (Figure 12) containing AgNPs were prepared for biomedical applications [154,155]. Further investigations revealed antibacterial properties of the fibers [156], which were used for the fabrication of antibacterial fabrics for wound dressing and other tissue engineering applications [155,157].

Anionic HYH was combined with cationic polymers, such as poly(dimethyldiallylammonium chloride) [158], chitosan [159,160,161], poly(L-lysine) [162,163], and poly(ethyleneimine) [164], for the fabrication of multilayer films by the LBL self-assembly method. AgNPs were incorporated into the films by in situ synthesis [158,164] or by co-deposition with polymers [159]. The pre-complexation of poly(l-lysine) with Ag^+^ ions before self-assembly resulted in agglomerated AgNPs, whereas post-loading of the multilayer films allowed for improved AgNP dispersion [163]. The composite films showed a good antimicrobial capability [158,159], which makes them promising for wound dressing applications [159]. The microstructure and properties of the films are influenced by different factors, such as the HYH and Ag concentration, the size of AgNPs, and the number of individual polymer layers [159]. It was also found that embedded AgNP arrays possess good stability for localized surface plasmon resonance absorption spectrum-based biosensors [158].

Another LBL self-assembly technique [165] involved the dispersion and surface modification of AgNPs with chitosan or aminocellulose, which were used for the co-deposition with HYH. This technique facilitated the fabrication of free-standing films for different applications to control bacterial growth on injured skin, burns, and chronic wounds [165].

### 5.2. Gold

The use of HYH–Au composites (Supplementary Materials, Table S1) has emerged as an interesting strategy, particularly in the field of biomaterial development and drug delivery [166]. Au nanoparticles (AuNPs) have attracted attention for modification of biomaterials because they have been shown to promote cell attachment and enhance proliferation [167]. Moreover, AuNPs protected HYH-based multilayer films from fast degradation [168]. The fabrication of HYH–Au composites facilitated the development of conceptually new biomedical techniques, such as laser welding of connective tissues and healing tissue defects [169]. In the investigation of HYH–poly(l-lysine) multilayer films [170], it was demonstrated that the elastic modulus of the films can be changed by more than one order of magnitude by the addition of AuNPs. The films are promising for the development of new techniques for controlled drug release applications due to the ability to remotely heat well-defined sites by laser irradiation [171]. The use of HYH-based multilayers [172] facilitated the in situ synthesis of AuNPs of controlled size. It was found that the carboxylic and hydroxyl groups of HYH played a key role in the formation of AuNPs [172].

### 5.3. Quantum Dots

HYH was loaded with semiconducting quantum dots (QDs) (Supplementary Materials, Table S1) to study scaffold angiogenesis and scaffold–tissue interactions [173,174]. The QDs were strongly luminescent at room temperature, and their emission wavelength was tuned from blue to red by changing the particle size [173]. QD-labeled collagen–HYH scaffolds were prepared by the freeze-drying method [173]. The scaffolds showed biocompatibility and degradability and were tested in animal implantation experiments. The QD-labeled collagen–HYH scaffold provides a promising tool to study scaffold–tissue interactions by 3D confocal microscopy [173]. Investigations highlighted the influence of the molecular size of HYH, porosity, and crosslinking on the performance of HYH scaffolds containing QDs [173,174].

HYH–QDs composites were prepared using LBL self-assembly for sensors, based on fluorescent properties of QDs, and for other applications [175]. CdS/HYH and ZnS/HYH QD thin-film biocomposites were prepared by in situ synthesis, and it was found that HYH is efficient in preventing the aggregation and controlling the growth of ZnS and CdS nanocrystals [176]. In this approach, HYH was used as a biocompatible capping agent. The electric charge of modified QDs was analyzed and it was found that charged particles can be used for the fabrication of ordered heterostructures. The ability to form patterned structures was beneficial for applications in biomedical devices [175]. Investigations demonstrated the fabrication of foils emitting light on excitation with a UV light. The wavelength of the emission was varied by the changing the QD size and concentration [176]. The composite films showed promising properties for applications in fluorescent labels and probes [176].

## 6. Conclusions and Future Trends

The literature data described in this review indicate that HYH gels loaded with HAP or CaP are promising materials for bone tissue regeneration and the treatment of bone and skin defects. Further progress in this area can be achieved by the optimization of the HYH molecular size, chemical modifications of HYH molecules, the development of advanced crosslinking techniques, and the optimization of the size and shape of HAP or CaP particles and their dispersion. Moreover, composite gels can be loaded with other functional materials for the fabrication of new composites with advanced functionality.

New film deposition mechanisms based on the pH-dependent charge and solubility of HYH as well as HYH adsorption on HAP are key factors for emerging developments in the field of surface modification of biomedical implants. Further advances can be achieved by the development of film crosslinking techniques to improve the chemical stability of the films. EPD offers the potential for co-deposition of HYH and HAP with other functional biomaterials. One of the remaining challenges in this area is related to charge reversal of HYH at a low pH and solubilization of HYH gel, which are detrimental to the formation of adherent films by anodic EPD.

Investigations demonstrated the promising performance of HYH–HAP and HYH–CaP scaffolds, which combine properties of organic and inorganic components. The primary research focus in this area is the development of composites containing other polymers and functional materials. Significant interest has been generated in the development of efficient crosslinkers, application of BCaP, microstructure control, and development of advanced fabrication techniques.

Many examples herein suggest that HYH-CPCs are promising materials for various biomedical applications. The most important advancements are related to the optimization of the composition of the inorganic CPC phase, modification of the HYH, and optimization of the microstructure and chemical interactions of CPC and HYH phases. Various properties have been optimized, such as mechanical properties, the setting time, the injectability, and the degradation rate. This fast-growing field is exciting on several applied and fundamental levels.

The synergy of functional properties of HYH and HAP/CaP offers a strong potential for applications of composite particles in drug delivery and tumor treatment. From the available literature, it is obvious that further development of fabrication procedures, particle microstructures, and compositions will result in new important advances in this field. Bioceramic particles with a HYH-modified surface chemistry have also been used to expand the functionality of HYH-based composites. The ability to modify a bioceramic’s surface chemistry opens up new opportunities for the fabrication of advanced scaffolds and materials for drug delivery. Of particular interest are applications of HYH as a biocompatible dispersing, capping, and structure-directing agent for the synthesis of bioceramic particles. The bioactivity of HYH provides a platform for advanced implant applications.

From the available literature, it is obvious that the development of material processing techniques will play an important role in the fabrication of HYH–AgNPs, HYH–AuNPs, and HYH–QDs composites. It is expected that further progress in this area will result in the development of advanced materials with antibacterial products for various biomedical applications and advanced biosensors.

## Data Availability

Data sharing is not applicable.

## Supplementary Materials

The following are available online at https://www.mdpi.com/article/10.3390/ma14174982/s1, Table S1: HYH composites, phase content, fabrication methods, performance and references.
