# Supplementary material for: Hyaluronic-Acid-Based Organic-Inorganic Composites for Biomedical Applications
Source: Materials (Basel). 2021 Aug 31;14(17):4982. doi: 10.3390/ma14174982 (PMC8434239; doi:10.3390/ma14174982)
Supplement: Supplementary file 1 [file materials-14-04982-s001.zip › materials-1348158-supplementary.pdf]

materials

Supplementary Materials

Hyaluronic-Acid-Based Organic–Inorganic Composites for Biomedical Applications

Rebecca Sikkema, Blanca Keohan and Igor Zhitomirsky *

Department of Materials Science and Engineering, McMaster University, Hamilton, ON L8S4L7, Canada; sikkemar@mcmaster.ca (R.S.); keohanb@mcmaster.ca (B.K.)
* Correspondence: zhitom@mcmaster.ca (I.Z.)

Citation: Sikkema, R.; Keohan, B.; Zhitomirsky, I. Hyaluronic-Acid-Based Organic–Inorganic Composites for Biomedical Applications. *Materials* **2021**, *14*, 4982. https://doi.org/10.3390/ma14174982

Academic Editor: Maja Dutour Sikirić

Received: 3 August 2021
Accepted: 27 August 2021
Published: 31 August 2021

Publisher's Note: MDPI stays neutral with regard to jurisdictional claims in published maps and institutional affiliations.

Copyright: © 2021 by the authors. Licensee MDPI, Basel, Switzerland. This article is an open access article distributed under the terms and conditions of the Creative Commons Attribution (CC BY) license (http://creativecommons.org/licenses/by/4.0/).

Table S1. HYH composites, phase content, fabrication methods, performance and references

Composite	Phase Content	Fabrication Method	Application	Performance	Reference
Gel	HYH-nanoHAP	Rapid mixing and injection	Bone formation	Improved biocompatibility	[22]
Gel	Bisphosphonate functionalized HYH-CaP	Mixing and non-covalent or covalent cross-linking	Filling bone defect; Bone regeneration	Rapid self-healing; Improved robustness and biodegradability of non-covalently crosslinked gels	[23]
Gel	Methacrylated gelatin-methacrylated HYH-HAP	Crosslinking and 3D printing	Bone 3D bioprinting	Excellent printability and structural integrity	[36]
Gel	Pullulan-HYH-HAP	Mixing and crosslinking	Dermal Rejuvenation	Improved storage modulus, viscosity and long-lasting durability	[37]
Gel	Glycidyl methacrylate conjugated HYH-nanoCaP	Photo-crosslinking and in situ precipitation	Soft tissue engineering	Improved mechanical behavior and biostability	[38]
Gel	Silk Fibroin-HYH-HAP	Mixing and crosslinking	Filling irregular bone defects	Improves cell growth and survival; increases mechanical strength	[39]
Gel	HYH-microHAP	Mixing and crosslinking	Soft tissue augmentation	Long-lasting durability; significant improvement in volumetric maintenance	[40]
Gel	Glycol chitosan-HYH-nanoHAP	Mixing via ultrasound	Bone tissue engineering	Excellent biocompatibility and biodegradability	[41]
Gel	Thiolated HYH-HAP	Mixing and crosslinking	Bone tissue engineering	Increase in cell viability and proliferation; improved physical properties	[42]
Gel	HYH-HAP-extracellular matrix microparticles	Mixing	Bone regeneration; bone and cartilage tissue engineering	Increased storage moduli, yield stress and viscoelastic recovery	[43]
Gel	HYH-nanoHAP	Crosslinking and in situ precipitation	Soft tissue augmentation	Great enhancement of dermal matrix; high stiffness and gel cohesiveness	[44]
Gel	HYH-nanoHAP-zolendronate	Mixing and crosslinking	Bone repair and augmentation	Induces rapid mineralization; inhibits resorption	[45]
Gel	Collagen-dimethylsilanediol HYH-nanoHAP-(polyethylenimine or arginine)	Mixing and crosslinking	Bone tissue engineering; gene carrier	Significant improvement in compressive modulus; high toughness	[46]
Gel	HYH-CaP	Photocrosslinking, in situ precipitation and ionic crosslinking	Hard tissue engineering	Enhanced bioactivity; enhanced mechanical and biological properties	[47]

Gel	Alginate-HYH-HAP-exosome	Mixing and crosslinking	Tissue and organ repair	Enhanced bone regeneration and mechanical strength; improved sustained exosome release	[48]
Gel	Chitosan-HYH-HAP	Mixing, crosslinking and irradiation	Drug delivery	Improved drug uptake and release capability	[49]
Gel	Gelatin-HYH-HAP-calcium sulfate	Mixing and crosslinking	Bone regeneration in bone defects	Enhanced bone regeneration; good biocompatibility and biodegradability	[50]
Gel	Pyrogallol conjugated HYH-HAP	Poured into mold, mixed and frozen	Orthopedics; bone tissue engineering	Increased osteoconductivity; improved mechanical strength	[51]
Film	HYH-HAP	Electrodeposition	Corrosion protection	High deposition rates; Control deposit composition and microstructure	[24]
Film	Bioglass-HYH-HAP	Electrophoretic deposition	Implant coating	Enhanced bioactivity	[55]
Film	Bioglass-HYH-nanoHAP	Electrophoretic deposition	Surface modification of implants; biosensor fabrication	Improved biocompatibility	[56]
Film	Halloysite nanotube-HYH-HA	Electrophoretic deposition	Biomedical implants; Drug delivery	Improved bioactivity and biocompatibility	[57]
Film	Multiwalled carbon nanotube-HYH-HAP	Electrophoretic deposition	Corrosion protection	Improved strength and toughness	[58]
Film	Graphene oxide-HYH-HAP	Anodic electrophoretic deposition	Corrosion protection	Increased deposition rate; Inhibited crack creation and propagation	[59]
Film	Chitosan-HYH-HAP-Cellulose nanocrystal	Layer-by-layer deposition	Bone scaffold	Increased bioaffinity and biocompatibility; improved surface hydrophilicity; enhanced mechanical properties	[60]
Film	Bone morphogenetic protein-2 – HYH-HAP	Adsorption	Implant coating	Increased bone growth	[61]
Film	Gantrez-poly(allylamine-co-allylguanidinium)-HYH-HAP	Adsorption	Antimicrobial coating	Significant decrease in bacterial viability	[62]
Film	Bone morphogenetic protein-2 – HYH-HAP	Adsorption	Implant coating	Improved osseointegration	[63]
Film	HYH-HAP	Adsorption	Bone engineering; Dental applications	Improved biocompatibility	[64]

Film	Chitosan-HYH-HAP	Sprayed	Antibacterial coatings	High antibacterial activity	[65]
Scaffold	Gelatin-HYH-CaP	Molding	Bone regeneration	Decreased swelling and degradation rates; increased cell growth and proliferation	[66]
Scaffold	Chitosan-Collagen-HYH-HAP	Freeze-drying, crosslinking and in situ precipitation	Tissue engineering; Regenerative medicine	Improved mechanical parameters, thermal stability and porosity	[67]
Scaffold	Alginate-HYH-HAP	Crosslinking, freeform 3D printing, crosslinking	Interface tissue engineering	Improved mechanical and physiological stability; Accelerated printing method for complex geometries	[68]
Scaffold	Chitosan-Collagen-HYH-CaP	Freeze-drying, crosslinking and in situ precipitation	Tissue engineering; Regenerative medicine	Improved biocompatibility, mechanical parameters, porosity and density	[69]
Scaffold	Collagen-chitosan-HYH oligosaccharide-HAP-poly(lactic-co-glycolic acid)	Crosslinking, freeze-drying and placed in a mold	Bone tissue engineering	High porosity and water uptake capacity	[70]
Scaffold	Polyvinyl alcohol-HYH-HAP	Physical crosslinking by freezing-thawing	Bone tissue engineering	Improved cell proliferation	[71]
Scaffold	Methoxy poly(ethylene glycol)-block-poly(Ɛ-caprolactone)-glycidyl methacrylate HYH-HAP	Fused deposition modeling method	Cartilage regeneration; osteoarthritis treatment	Significantly increased compressive strength	[72]
Scaffold	Gelatin-HYH-CaP	Sponge replica method, sintering, dropwise polymer addition, freeze-drying and crosslinking	Bone regeneration	High interconnected porosity; increase in cell proliferation	[73]
Scaffold	Poly(γ-glutamic)-HYH-nanoHAP	Molding	Stem cell differentiation; osteochondral tissue engineering	Good toughness, strength, viscoelasticity, and cytocompatibility	[74]
Scaffold	Chitosan-chondroitin sulfate-HYH-HAP	In situ biomimetic synthesis and freeze-drying	Bone tissue engineering	Good biocompatibility; enhanced osteoblast proliferation and differentiation	[75]
Scaffold	Chitosan-collagen-HYH-nanoHAP	Freeze-drying	Regenerative medicine	Improved cell attachment and growth; high porosity	[76]
Scaffold	Chitosan-HYH-nanoHAP	Crosslinking, frozen and lyophilized	Bone repair	Excellent cell penetration, growth and proliferation	[77]
Scaffold	Atelocollagen-HYH-HAP-CaP	Chondral phase: crosslinking and molded; Osseous	Osteochondral defect repair	High strength and stiffness	[78]

		phase: urethane foaming method and sintered			
Scaffold	Chitosan-collagen-HYH-HAP	Crosslinking, frozen and lyophilized	Bone regeneration	Improved mechanical parameters; enhanced biological properties; good biocompatibility	[79]
Scaffold	Chitosan-collagen-HYH-nanoHAP	Crosslinking and freeze-drying	Tissue regeneration	Good biocompatibility; highly porous; good stability	[80]
Scaffold	Gelatin-HYH-HAP	Freeze-drying and crosslinking	Dental applications	Excellent hemostatic properties; high rate of collagen distribution	[81]
Scaffold	Chitosan-HYH-CaP-bone morphogenetic protein-2	BCP formation: Sponge replica method, sintered; Polymer: crosslinked, molding	Bone tissue regeneration	Significantly improved mechanical strength; highly porous	[82]
Biocement	Platelet lysate-HYH-CaP	Crosslinking and mixing, self-setting	Alveolar bone regeneration	Improved biocompatibility	[83]
Biocement	Chitosan-HYH-HAP	Mixing powder and liquid phases	Bone tissue engineering	Good biocompatibility	[84]
Biocement	Citric acid-HYH-CaP	Mixing of powder and liquid phases	Filling bone defects	Good cohesion, compressive strength and sufficient shelf life	[85]
Biocement	Calcium sulfate hemihydrate-HYH-collagenase-HAP	Mixing of powder and liquid phases	Alveolar bone regeneration	Excellent biocompatibility; improved bone formation	[86]
Biocement	Platelet lysate-HYH-CaP	Mixing of powder and liquid phases	Bone regeneration	Improved mechanical properties, degradability and biological performance	[87]
Biocement	CAP-HYH	Mixing of powder and liquid phases	Filling bone defects	Improved cement cohesion	[88]
Biocement	CAP-HYH	Mixing of powder and liquid phases	Injectable bone filler material	Improved cement injectability, improved compressive strength	[89]
Biocement	CAP-HYH, citric acid additive	Mixing of powder and liquid phases	Bone defects healing	Enhanced compressive strength, improved osteoinductivity	[90]
Biocement	HYH-alginate beads-CAP	Synthesis by a spraying method	Injectable bone substitute for improved bone regeneration	Increased injectability and washout resistance	[91]
Biocement	CaP-citric acid-sodium bicarbonate-HYNa	Mixing of powder and liquid phases	Bone repair or reconstruction	Enhanced bioactivity	[92]
Biocement	CAP-HYH-thiol modified with bisphosphonate	Mixing of powder and liquid phases	Bone repair	Enhanced cohesion	[93]

Biocement	CaP-HYH-tyramine	Mixing of powder and liquid phases	Healing of small bone and joint defects	Enhanced mechanical strength	[94]
Composite particles	CaP-HYH, HAP-HYH	Self-assembly and crosslinking	Melanoma tumor therapy	Enhanced stability, selective release of functional biomolecules	[95]
Composite particles	Oligo-HYH-silica-HAP	Multistep nanoparticle formation and surface modification	Targeted drug delivery	Enhance drug loading, improved pH-responsiveness for drug release	[96]
Composite particles	HAP-HYH	Biomineralization method	Drug delivery	Improved drug release rate	[97]
Composite particles	Polyethyleneimine stabilized HAP-HYH	Chemical precipitation and surface modification	Targeted drug delivery	Enhanced dispersibility, enhanced stability, improved drug release efficiency	[98]
Composite particles	CaP-dopa-HYH conjugate	Chemical precipitation and surface modification	Gene delivery	Improved stability and enhanced delivery efficiency	[99]
Composite particles	HAP-HYH	Surface modification of HAP	Targeted drug delivery	Improved tumor targeting, enhanced delivery efficiency	[100]
Composite particles	HYH-doped HAP	Biomimetic synthesis and functionalization	Tumor-specific bioimaging	Enhanced imaging performance	[101]
Composite particles	HYH-CaP	Chemical precipitation and surface modification	Tumor targeted therapy	Improved si-RNA delivery	[102]
Composite particles	Hollow-HAP-HYH-chitosan	Surface modification, hydrothermal synthesis, chemical etching, self-assembly	Targeted drug delivery	Improved pH-dependent drug delivery properties	[103]
Composite particles	HAP-HYH-chitosan capped with Au nanoparticles	Chemical precipitation and surface modification	Remotely triggered drug delivery	Excellent near-infrared-triggered drug release property	[104]
Composite particles	HYH/polyethylene glycol-nano-HAP	Chemical precipitation and surface modification	Drug delivery	High drug loading efficiency	[105]
Composite gels	HYH -silica	Sol-gel method	Soft and hard tissue repair	Enhanced mechanical properties, improved bioactivity, high degradation resistance	[106]
Composite gels	HYH -silica	Sol-gel method	Tissue engineering	Enhanced thermal stability	[107]

Composite gels	HYH-chitosan-silica	Sol-gel method	Tissue engineering	Enhanced biocompatibility	[108]
Composite gels	HYH -silica	Chemical precipitation and surface modification	Tissue engineering	Improved mechanical properties	[109]
Composite gels	HYH-silica	Chemical precipitation and surface modification	Biosensors	Enhanced electrochemiluminescence signal for the HYHase detection	[110]
Composite gels	HYH-nPSi	Solution processing	Drug delivery	Improved stability and control of drug release	[111]
Composite gels	HYH-silica-Au	Solution processing and surface modification	Drug delivery for cancer treatment	Improved control of drug release	[112]
Composite gels	HYH-silica	Colloidal processing and freeze-drying	Wound healing	Improved healing	[113]
Film	HYH-silica	Electrophoretic deposition	Surface modification of implants	Improved adhesion and biocompatibility	[114]
Coating	HYH-silica	Sol-gel and electrophoretic deposition	Surface modification of implants	Enhanced biolubrication properties	[115]
Film	HYH - Poly (6-lysine)-silica	Solution processing	Free standing membranes	Improved film stiffness and biocompatibility	[116]
Film	HYH-silica	Layer-by-layer deposition	Drug delivery	Enhanced drug loading	[117]
Film	HYH-silica	Layer-by-layer deposition	Drug delivery	Controlled drug release speed	[118]
Coating	HYH-collagen-titania nanotubes	Anodization, drop coating	Drug delivery	Sustained drug release, osseointegration effect	[119]
Coating	Methacrylated HYH-titania nanofibers	Electrospinning, drop casting	Orthopedic implants	Improved biocompatibility	[120]
Coating	Titania nanotubes modified with chitosan and alendronate grafted HYH	Spin-assisted layer-by-layer coating	Drug delivery	Enhanced osseointegration	[121]
Coating	Titania nanotubes-HYH-chitosan	Layer-by-layer deposition	Orthopedic implants	Enhanced antibacterial properties, improved drug delivery	[122]
Coating	Titania nanotubes-dopamine modified HYH-chitosan	Anodization, Layer-by-layer deposition	Orthopedic implants	Enhanced antibacterial properties, enhanced osseointegration	[123]
Composite particles	HYH-titania	Sol-gel	Tissue engineering	Antifungal activity	[124]
Composite particles	HYH-titania	Solution processing	Tissue engineering	Anticancer activity	[125]

Coating	HYH-titania-carbon dot-Cu^{2+}	Solution processing	Biosensor	Cancer diagnosis, antifouling effect	[126]
Scaffold	HYH-$CaCO_3$	Solution impregnation	Orthopedic implants	Enhanced bone regeneration	[127]
Scaffold	HYH-chitosan--$CaCO_3$	Solution processing	Orthopedic implants	Enhanced mechanical properties, improved cell proliferation	[129]
Composite particles	HYH-$CaCO_3$	Colloidal processing	Particles for protein delivery	Enhanced protein delivery efficiency	[131]
Composite particles	HYH-alumina	Chemical precipitation and colloidal processing	Liver cancer therapy	Enhanced drug delivery efficiency	[132]
Composite membrane	HYH-alumina	Solution processing	Immunosensor for detection of bacterial pathogens	Efficient pathogens detection	[133]
Composite gel	HYH-zirconia	Solution processing	Implant with controlled release of growth factors or proteins	Improved cell proliferation and differentiation	[134]
Scaffold	HYH-gelatin-bioglass	Sol-gel and freeze-drying	Bone tissue engineering	Improved biocompatibility and bioactivity	[135]
Scaffold	HYH-collagen-bioglass	Solution processing	Bone tissue engineering	Improved biocompatibility and bioactivity	[136]
Injectable biocomposite	HYH- bioglass	Colloidal processing	Bone tissue engineering	Improved biocompatibility and bioactivity	[137]
Coating	HYH–dopamine-conjugate-Ag-doped bioglass	Layer-by-layer deposition	Bone tissue engineering	Enhanced bioactivity and bactericidal properties	[138]
Coating	HYH-catechol functionalized chitosan-bioglass	Dip-coating	Orthopedic implants	Improved biocompatibility and bioactivity	[139]
Film	HYH-catechol functionalized chitosan-bioglass	Spin-coating layer-by-layer deposition	Hard tissue regeneration	Improved biocompatibility and bioactivity, improved adhesion, reduced swelling	[140]
Film	HYH-DOPA-Chitosan-silver-doped bioglass	Layer-by-layer deposition	Bone regeneration	Improved biocompatibility bioactivity, and antibacterial properties	[141]
Composite gel	Biphosphonate modified HYH-bioglass	Solution and colloidal processing	Bone regeneration	Self-healing and bioactive processing	[142]
Scaffold	HYH-bioglass	Solution and colloidal processing	Bone regeneration	Improved biocompatibility and bioactivity	[143]
Composite particles	HYH-bioglass	Sol-gel	Bioactive paste for bone regeneration	Improved bioactivity	[144]
Composite paper	HYH-curcumin-Ag-chitosan	Solution and colloidal processing	Antibacterial paper	Enhanced antibacterial properties	[145]
Composite sponge	HYH-chitosan-l-glutamic acid-Ag	Solution and colloidal processing	Antibacterial sponge	Enhance wound-healing activity	[146]

Composite hydrogel	HYH-corn silk-Ag	Microwave-assisted synthesis and solution processing	Wound healing	Enhanced wound-healing activity and antimicrobial properties	[147]
Composite particles	HYH-Ag	Solution and colloidal processing	Wound healing	Enhanced wound-healing activity and antimicrobial properties	[148]
Composite sponge	HYH-chitosan-Ag	Colloidal processing and freeze-drying	Wound dressing	Enhanced antibacterial properties	[149]
Composite gel	HYH-alginate-Ag	Solution and colloidal processing	Wound dressing	Enhanced antibacterial properties	[150]
Composite membrane	HYH-polycaprolactone-Ag	Solution and colloidal processing	Healing after tendon surgery	Prevention of peritendinous adhesion	[151]
Composite films	HYH-lecithin-Ag	Solution and colloidal processing	Wound dressing	Enhanced antibacterial properties	[152,153]
Composite fibers and fabrics	HYH-Ag	Electrospinning and colloidal processing	Wound dressing	Enhanced biocompatibility and antibacterial properties	[154-156]
Composite fibers and fabrics	HYH-polyvinyl alcohol-Ag	Electrospinning and colloidal processing	Skin regeneration and drug delivery	Enhanced biocompatibility, antibacterial and drug delivery properties	[157]
Film	HYH-PDDA-Ag	Layer-by-layer deposition	Antimicrobial applications	Enhanced antimicrobial properties	[158]
Composite fabric	HYH-chitosan-Ag	Layer-by-layer deposition and colloidal processing	Wound dressing	Enhanced antimicrobial properties	[159]
Coating	HYH-chitosan-Ag	Solution synthesis and colloidal processing	Antibacterial surface development	Enhanced antibacterial properties	[160]
Film	HYH-chitosan-Ag	Layer-by-layer deposition and colloidal processing	Antibacterial surface development	Enhanced antibacterial properties	[161]
Film	HYH-poly-l-lysine-Ag	Layer-by-layer deposition and colloidal processing	Antibacterial surface development	Enhanced antibacterial properties	[162]
Free standing film	HYH-poly-l-lysine-Ag	Layer-by-layer deposition and colloidal processing	Wound dressing	Enhanced antibacterial properties	[163]
Film	HYH-polyethylenimine-Ag	Layer-by-layer deposition and colloidal processing	Immunoassay application	Improved fluorescent signal	[164]
Film	HYH-chitosan-Ag	Layer-by-layer deposition and colloidal processing	Wound dressing	Enhanced antibacterial properties	[165]
Injectable hydrogel	HYH-polyethylene glycol-Au	Solution and colloidal processing	Treatment of heart disease	Enhanced biocompatibility	[166]
Nanofiber meshes	HYH-collagen-Au	Electrospinning and colloidal processing	Tissue engineering	Enhanced biocompatibility	[167]
Composite particles	HYH-polylysine-Au	Solution and colloidal processing	Tissue engineering	Improved cellular adhesion and biocompatibility	[168]

Composite particles	HYH-Au nanorod	Solution synthesis and colloidal processing	Diagnosis, imaging and therapy of cancer	Advanced optical and absorption properties	[169]
Film	HYH-poly-l-lysine-Au	Solution synthesis and colloidal processing	Cell culture applications	Enhanced film stiffness, enhanced cell adhesion	[170]
Film	HYH-poly-l-lysine-Au	Layer-by-layer deposition and colloidal processing	Implant biocoating, tissue engineering	Enhanced near-IR absorption, biocompatibility	[171]
Film	HYH-poly-l-lysine-Au	Layer-by-layer deposition and colloidal processing	Biosensors, drug delivery	Improved biocompatibility, homogeneous composition, drug storage ability	[172]
Scaffold	HYH-collagen-CdSe/ZnS quantum dot	Solution synthesis and colloidal processing, freeze drying	Biosensors	Enhanced biocompatibility	[173]
Scaffold	HYH-collagen-quantum dot	Solution synthesis and colloidal processing	Tissue engineering	Enhanced biocompatibility and fluorescence	[174]
Film	HYH-ZnS/CdSe quantum dot	Layer-by-layer deposition and colloidal processing	Sensors	Enhanced biocompatibility and fluorescence	[175]
Film	HYH-ZnS and HYH-CdSe quantum dots	Colloidal processing	Fluorescent labels and probes	Enhanced biocompatibility and fluorescence	[176]
